# Characterizing Metabolic Shifts in Septic Murine Kidney Tissue Using 2P-FLIM for Early Sepsis Detection

**DOI:** 10.3390/bioengineering12020170

**Published:** 2025-02-10

**Authors:** Stella Greiner, Mahyasadat Ebrahimi, Marko Rodewald, Annett Urbanek, Tobias Meyer-Zedler, Michael Schmitt, Ute Neugebauer, Jürgen Popp

**Affiliations:** 1 Leibniz Institute of Photonic Technology (Leibniz IPHT), Member of the Research Alliance “Leibniz Health Technologies”, Member of the Leibniz Center for Photonics in Infection Research (LPI) Jena, Albert-Einstein-Straße 9, 07745 Jena, Germany; stella.greiner@leibniz-ipht.de (S.G.); mahyasadat.ebrahimi@leibniz-ipht.de (M.E.); annett.urbanek@leibniz-ipht.de (A.U.); tobias.meyer@leibniz-ipht.de (T.M.-Z.); m.schmitt@uni-jena.de (M.S.); ute.neugebauer@ipht-jena.de (U.N.); 2 Institute of Physical Chemistry and Abbe Center of Photonics, Friedrich Schiller University Jena, Helmholtzweg 4, 07743 Jena, Germany; 3 Dipartimento di Fisica, Politecnico di Milano, Piazza Leonardo da Vinci 32, 20133 Milano, Italy; marko.rodewald@polimi.it; 4 Center for Sepsis Control and Care and Department of Anaesthesiology and Intensive Care Medicine, Jena University Hospital, Am Klinikum 1, 07747 Jena, Germany

**Keywords:** metabolic non-linear imaging, FLIM, sepsis, kidney

## Abstract

In this study, thin mouse kidney sections from healthy mice and those infected leading to acute and chronic sepsis were examined with two-photon excited fluorescence lifetime imaging (2P-FLIM) using the endogenous fluorescent coenzymes nicotinamide adenine dinucleotide (NADH) and flavin adenine dinucleotide (FAD). The results presented show that this approach is a powerful tool for investigating cell metabolism in thin tissue sections. An adapted measurement routine was established for these samples by performing a spectral scan, identifying a combination of two excitation wavelengths and two detection ranges suitable for detailed scan images of NADH and FAD. Selected positions in thin slices of the renal cortex of nine mice (three healthy, three with chronic sepsis, and three with acute sepsis) were studied using 2P-FLIM. In addition, overview images were obtained using two-photon excited fluorescence (2PEF) intensity. This study shows that healthy kidney slices differ considerably from those with acute sepsis with regard to their fluorescence lifetime signatures. The latter shows a difference in metabolism between the inner and outer cortex, indicating that outer cortical tubular cells switch their metabolism from oxidative phosphorylation to glycolysis in kidneys from mice with acute sepsis and back in later stages, as seen for mice with chronic infections. These findings suggest that 2P-FLIM could serve as a powerful tool for early-stage sepsis diagnosis and monitoring metabolic recovery during treatment.

## 1. Introduction

In cases where an infection encounters a dysregulated host response, this can lead to the failure of one or more organs. This phenomenon is described as sepsis, a disease with a high mortality rate [1,2]. In 2017, there were 49 million cases of sepsis worldwide, resulting in 11 million deaths. This represents 20% of global deaths, emphasizing the importance of studying this disease [3]. The World Health Organization has identified the prevention, diagnosis and clinical treatment of sepsis as a priority area in a resolution published in 2017 [4]. Biophotonic imaging using predominantly spectroscopic imaging approaches is a promising tool for the study of such diseases, with the potential to improve our understanding and facilitate the development of rapid and effective treatments [5,6]. Compared to other imaging modalities, FLIM provides spatially resolved insights into metabolic processes without the need for exogenous markers, which is crucial for understanding dynamic changes in sepsis pathology. In this context, fluorescence imaging using one- or multi-photon excitation schemes is particularly noteworthy [7,8]. In addition to the fluorescence intensity, the fluorescence lifetime is especially susceptible to change in response to small changes in the chemical environment [9]. The advantages of two-photon excited fluorescence lifetime imaging (2P-FLIM) over single-photon excited 1P-FLIM include the elimination of overlap between excitation and emission spectra and the temporal and spatial concentration of fluorescence in the focal plane [10]. Metabolic FLIM does not require the introduction of an exogenous fluorescent label because it is based on the fluorescence of two metabolic autofluorophores, nicotinamide adenine dinucleotide (NADH) and flavin adenine dinucleotide (FAD), in their protein-bound and free forms, which have different lifetimes [11,12]. This differentiation is essential, as it allows for the precise monitoring of metabolic shifts from oxidative phosphorylation to glycolysis, which are hallmark processes in sepsis-induced tissue damage. Thus, 2P-FLIM represents a promising tool for sepsis research, as briefly summarized in Table 1.

In order to observe NADH and FAD independently, it is necessary to use appropriate excitation and detection wavelengths due to the spectral overlap in the excitation and emission spectra of NADH and FAD [13,14,15]. Previous studies have reported varying fluorescence lifetime properties for NADH and FAD in studying various media (different types of cells and tissues) while applying varying excitation and detection wavelengths [8,14,16,17,18,19,20]. As a result, a spectral FLIM scan of the tissue is advisable prior to detailed measurements. The ratio of the relative amplitudes of the bound fluorophores (fluorescence lifetime imaging redox ratio, FLIRR) gives insight into the metabolism of the tissue under investigation and summarizes the measurements at one location into a single value [21]. A reductive metabolic pathway via glycolysis results in a lower FLIRR than that observed for oxidative phosphorylation. This parameter has already been established for the detection of cancerous tissue and is promising for use in infectious disease diagnostics such as sepsis, as well [22]. Because of the filter function of the kidney, sepsis often results in acute kidney injury (AKI) [23,24]. This is the rationale behind the selection of thin murine kidney sections as the specimen for this study. To gain insight into the diverse metabolic responses, healthy mice and mice with chronic and acute sepsis were studied using metabolic FLIM imaging.

## 2. Materials and Methods

### 2.1. Animal Handling and Thin Section Preparation

Polymicrobial sepsis was induced in mice by intraperitoneal injection of a slurry suspension of human feces (peritoneal contamination and infection (PCI) model [25]), and their behavior in the following hours up to 14 days was examined and scored. The scoring sheet reflects body weight, general condition, activity and reaction to external stimuli (see Table S1 in the Supplementary Material for details, adapted from ref. [24]). The resulting scores are shown in Table 2. Healthy mice from the non-infected control group were scored every three days. The chronic and acute mice were sacrificed (perfusion following anesthetization with 3.5% isoflurane) 24 h and 14 days after sepsis induction, respectively. All mice were housed under standardized conditions with controlled diet and environment to minimize external factors influencing metabolic responses.

The right kidney was extracted and shock-frozen. Frozen kidneys were cut into 20 µm-thin sections on a cryostat and thawed in nitrogen flow before refreezing and storing at −80 °C under nitrogen atmosphere. Prior to the measurements, samples were thawed under nitrogen atmosphere and measured in room air. All the experiments were conducted in accordance with approved ethical guidelines (see the section on the Institutional Review Board Statement).

### 2.2. Measurement Setup

A DeltaEmerald S09670 (APE, Berlin, Germany) laser was employed with a tuneable pump beam that was set to 711, 798 or 876 nm with a second laser at 1032.6 nm for overview images with temporal and spatial overlap of pulses.

Images were obtained using an inverse laser scanning microscope (Leica Microsystems, SP8 Falcon, Mannheim, Germany) equipped with a HC PL APO CS2 20×/0.75 NA dry objective. The fluorescence signal was collected using a hybrid photon detector (R10467U-40, Hamamatsu, Leica Microsystems, SP8 Falcon, Mannheim, Germany, counting mode) in an adjustable wavelength range in the epi direction, with the excitation light separated from the signal by an MFP SP665 and MP1 SP680 filter (both Leica Microsystems, SP8 Falcon, Mannheim, Germany). Furthermore, the FLIM signal of the detailed images was transmitted to a SPC-180NX S/N 3H0066 card (Becker & Hickl, Berlin, Germany).

To obtain overview images, rectangular multi-tile images with 10% overlap were acquired. Detailed images were obtained using 500 frame accumulations. The laser power was set to cause an initial photon count rate of 250 kcps for all measurements. Three positions each in the outer and inner cortex of the sample were measured, first with 876 nm excitation and 550–650 nm detection, and then with 711 nm excitation and 380–450 nm detection. A preliminary scan was performed with minimal laser power to identify potential measurement locations, followed by the acquisition of images at these locations and an overview image. Two spectral scans were performed on a healthy sample at one position in the cortex, first with excitation at 876 nm, Δλ = 0.7 nm and 53 mW (250 kcps at 550–650 nm) and a second with excitation at 711 nm, Δλ = 0.7 nm and 53 mW (200 kcps at 380–450 nm). The 10 nm-wide detection window was shifted in 57 steps of 5 nm within a total detection window of 380–670 nm. For each step, 20 frames were accumulated and two of these stacks were measured in succession for each excitation wavelength.

For imaging details, see also Appendix A.

## 3. Results

In this study, the effect of sepsis on the metabolism of murine kidneys was studied at two different time points: 24 h (acute, three mice: A1, A2, A3) and 14 days (chronic, three mice: C1, C2, C3) after sepsis induction. Three healthy mice (H1, H2, H3) were used as a control group. First, we present the changes in behavioral scores associated with sepsis. Secondly, we describe the different parts of murine kidney sections using a broad detection window in 2P-FLIM with a tile scanning overview. Based on these findings, positions for detailed FLIM images were chosen. We then performed a spectral FLIM scan to decide on suitable detection ranges for NADH and FAD. Finally, these ranges were used to obtain detailed FLIM images for thin sections of the nine mice at the chosen locations in the inner (i1, i2, i3) and outer renal cortex (o1, o2, o3).

### 3.1. Behavioral Scores

Table 2 shows the behavior of nine mice, of which six were infected to induce sepsis. Acute sepsis displays an early infection state. These mice were sacrificed 24 h after the infection. The scores at this time point vary greatly; one mouse showed no behavioral abnormalities (a score of 0, see Table 2) despite the infection. This difference in the biological immune answer can be attributed to biological variance. Chronic infection is the classification of mice sacrificed 14 days after the infection. In this time frame, in all animals, an initial rise of the score followed by a decrease can be observed (see mice C1, C2, C3 in Table 2), indicating a recovery.

### 3.2. Mean Photon Arrival Time Overview Images

**Figure 1:**
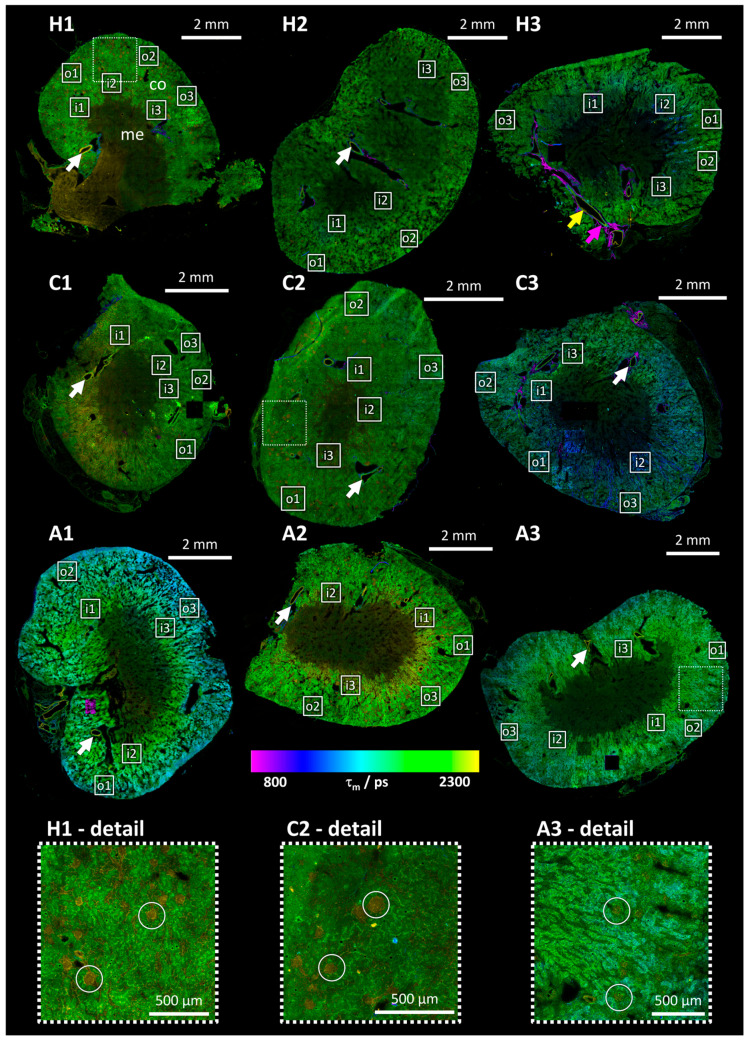
Pseudo-color mean photon arrival time overview images of murine kidney tissue thin sections of healthy (H1, H2, H3), chronically (C1, C2, C3) and acutely septic (A1, A2, A3) mice show a variety of structures. The labeled squares indicate the location of previously measured detail images (o1, o2, o3: outer cortex positions; i1, i2, i3: inner cortex positions, described and discussed in Section 3.4). The hue describes the mean photon arrival times (excitation 798 nm, detection 455–510 nm), whereas the brightness displays the detected photon number and was scaled for visibility on a per-image basis. The fluorescence intensity is higher in the outer region of the kidney, the renal cortex (co in sample H1), than in the inner one, the renal medulla (me in sample H1). The tiles missing for example in samples C1 and A3 are a result of photon saturation of the hybrid detector. Dashed squares indicate the position of the details of samples H1, C2 and A3 shown below. In these detailed images, the proximal and distal tubules are distinguishable by their mean photon arrival times with proximal tubules showing higher mean photon arrival times indicated by circles. Arrows indicate blood vessels, with colored arrows indicating structures in blood vessels with lower (purple) and higher (yellow) mean photon arrival times as a result of elevated collagen cross-linking in those areas with higher fluorescence lifetimes.

Mean photon arrival time overview images of murine kidney tissue sections (see Figure 1) provide an efficient and rapid overview of the respective sample, facilitating the localization of important structures while limiting the overall exposure and thus the risk of inducing photodamage. While there is observable fluorescence in the renal cortex (labeled “co” in Figure 1, sample H1), the renal medulla (labeled “me” in Figure 1, sample H1) exhibits minimal to no autofluorescence. It is thus possible to distinguish between these two main parts of the kidney and to localize the positions of detail measurements on the outer and inner border of the cortex, as illustrated by the labeled white squares in Figure 1. In comparison to human kidneys, the cortex is more pronounced in murine kidneys, occupying at least half of the section in these images [26,27].

These images demonstrate no notable distinction between the mean photon arrival times in healthy and chronic samples (see samples H1, H2, H3 (healthy) and C1, C2, C3 (chronic) in Figure 1). In the acute samples, the outer cortex exhibits a lower mean photon arrival time than the inner cortex (see samples A1, A2, A3 in Figure 1). This feature is exclusive in acute samples, hinting at a change in the metabolism specifically in the outer cortex at an early time point of the infection that is reverted later for chronic infections.

The fluorescence intensity in these overview FLIM images (see Figure 1) demonstrates that only in the cortex is there a sufficient number of photons for a two-component fit, which accounts for protein-bound and unbound NADH and FAD. The overall photon count is insufficient for a two-component fit in the entire overview image (especially the medulla; see, for example, the area marked with “me” in sample H1 in Figure 1), due to the low frame accumulation of 12 frames. Three positions were selected in each outer and inner cortex for detailed FLIM images with 500 frame accumulations for each position. These are indicated by white squares in Figure 1 and will be presented in Section 3.4.

The fluorescence lifetimes of the blood vessels, which can be identified by their morphology, differ from those of the surrounding tissue (indicated by arrows in sample H3 in Figure 1). These structures exhibit high fluorescence lifetimes (see yellow arrow in sample H3 in Figure 1), which can be attributed to elastin and collagen. [28,29]. In contrast, the outer part of the blood vessels exhibits very low mean photon arrival times (see purple arrow in sample H3 in Figure 1). This may be correlated with the degree of collagen cross-linking present within the tissue. The presence of cross-linking results in an elevated fluorescence lifetime [28]. While these features are prominent in the images (see arrows in Figure 1), their elevated collagen content exceeds the NADH/FAD model’s applicability, necessitating the avoidance of such positions in detailed measurements.

Another notable feature of the tissue is the presence of regions exhibiting elevated mean photon arrival times, which are discernible in multiple thin sections of the kidney (see circles in Figure 1). These regions are identified as proximal tubules, which exhibit distinct functional and metabolic characteristics compared to their distal tubule counterparts surrounding them. These differences are also reflected in their fluorescence properties [30]. Distal and proximal tubules represent the two main structures in the renal cortex and are therefore the only possible structures that these areas can be attributed to.

### 3.3. Spectral Scan

A spectral scan was performed on a healthy sample (mouse H2, parallel section to the one studied in other sections) to examine the fluorescence behavior at two different excitations and several ranges of detection wavelengths. The decay curves of all pixels in the FLIM image were aggregated and fitted to a bi-exponential decay curve (as shown in Equation (1)). Hereby, the weighted sum (using the amplitudes $a_{1}^{*}$ and $a_{2}^{*}$) of the mono-exponential decay curves of two fluorophores (indices 1 and 2) with the fluorescence lifetime ($\tau_{1}$, $\tau_{2}$) is convolved using an instrument response function (IRF). Using a measured IRF, the influence of the laser width and other characteristics of the optical setup can be considered (see Equation (1)).

(1) $$I=IRF*\left(a_{1}^{*}e^{-\frac{t}{\tau_{1}}}+a_{2}^{*}e^{-\frac{t}{\tau_{2}}}\right)$$

**Figure 2:**
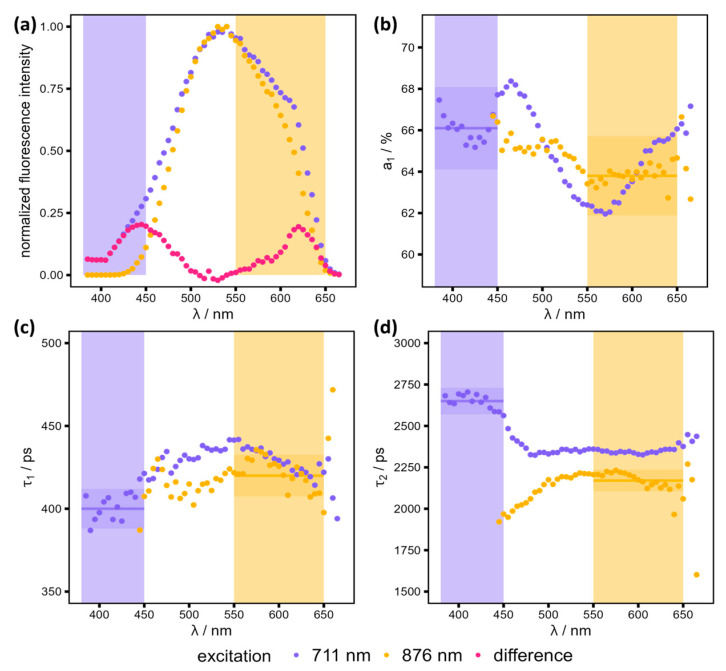
Determination of optimal excitation and detection wavelengths. Spectral FLIM scan of a healthy sample (mice H2 in bins of 10 frames for two excitation wavelengths (711 nm in violet, 876 nm in orange). The normalized fluorescence intensity is shown in different detection ranges for the two excitation wavelengths and the difference between the two (711–876, pink) (**a**), together with the behavior of the relative amplitude $a_{1}$. (**b**) and the lifetimes $\tau_{1}$ (**c**) and $\tau_{2}$ (**d**). Adequate detection ranges (violet (NADH) and orange (FAD) rectangles) were chosen regarding sufficient fluorescence intensity and minimal variance in the fitted lifetimes and amplitudes. The mean values of the fluorescence lifetimes and relative amplitudes in the chosen detection ranges are shown as horizontal lines with a 3% deviation indicated by a rectangle showing that almost all relevant data points lay in this range. Outliers are accepted to achieve a sufficient number of detected photons. The mean values are additionally listed in Table 3, and the lifetimes were used as fixed parameters in the pixel-wise fit of the detailed images described in Section 3.4.

Instead of the absolute amplitudes $a_{1}^{*}$ and $a_{2}^{*}$, the relative amplitude $a_{1}=\frac{a_{1}^{*}}{a_{1}^{*}+a_{2}^{*}}$ is used because it summarizes both amplitudes independent of the absolute fluorescence intensity. The resulting parameters of the two-component fit are presented alongside the normalized fluorescence intensity in Figure 2. This information was used to identify suitable detection windows for the distinct detection of NADH and FAD, which is critical due to their overlapping excitation and emission spectra [12].

Two-photon excitation of both NADH and FAD is achieved at a wavelength of 711 nm, resulting in the promotion of these molecules to an excited state [13,14]. It is therefore essential to select a suitable detection range to obtain lifetime information for NADH alone. The emission of NADH is blue shifted compared to FAD. Therefore, a lower wavelength window with constant lifetimes and amplitudes should be selected, considering the fluorescence intensity in order to obtain a sufficiently large detection window for a reasonable measurement time. For both excitation wavelengths (876 nm and 711 nm), the recorded emission maximum of the sample was approximately 530 nm (see Figure 2a). This corresponds to the emission maximum of FAD, indicating that the sample contains a greater amount of FAD than NADH. This is because FAD is excited by both excitation wavelengths, whereas NADH is only excited by the lower wavelength of 711 nm. The difference between the two normalized spectra in the region below 500 nm is analogous to the NADH emission spectrum (see Figure 2a) [13]. To minimize the FAD signal in the NADH channel, a detection window of 380–450 nm was selected for NADH, as illustrated by the purple rectangle in Figure 2. The lifetimes $\tau_{1}$ and $\tau_{2}$, as well as the relative amplitude $a_{1}$, remain largely constant within this region (see mean values as lines and rectangles showing 3% deviation in Figure 2b–d). The observed variation can be attributed to the relatively low fluorescence intensity and the excitation of a different fluorophore that exhibits a maximum fluorescence intensity around 625 nm as visible in the pink difference spectrum in Figure 2a. This fluorophore is most likely responsible for the difference in $\tau_{2}$ between the two excitation wavelengths and the rise in $a_{1}$ above 575 nm for an excitation wavelength of 711 nm (see Figure 2b,d). It does not interfere with the measurement of NADH because its emission spectrum does not overlap with the one of NADH.

For FAD, an excitation wavelength of 876 nm is already sufficient to separate the FAD signal from the NADH signal. A detection window of 550–650 nm is appropriate for achieving a reasonable photon count rate for the FLIM measurements. The differences in the lifetimes $\tau_{1}$ and $\tau_{2}$ and the relative amplitude $a_{1}$ between the two excitation wavelengths between 550 and 650 nm (see Figure 2) can be attributed to the excitation of another fluorophore that is excited using 711 nm. This is evident in the emission spectrum, which cannot be described with the two-component fit that was used.

Table 3 summarizes the chosen excitation and detection wavelengths for NADH and FAD, completed with approximate lifetimes and relative amplitudes that are, despite some small deviations, still in a similar wavelength range as values reported in the literature [20,31]. As fluorescence lifetime parameters are very prone to change with changes in the chemical environment, a change to these between different models—from isolated molecules to single cells to organs—is likely. As long as the only fluorophores registered are protein-bound/unbound FAD and NADH, only a change of $a_{1}$ is expected, while the lifetimes $\tau_{1}$ and $\tau_{2}$ remain constant. This is the reason why the lifetimes were fixed at the values given in Table 3 for the pixel-wise fit of the detailed images presented in Section 3.4.

### 3.4. Detailed FLIM Images

Detailed images acquired subsequently for FAD and NADH were fit pixel-wise to Equation (1) with the fixed lifetimes resulting from the spectral scan in order to obtain relative amplitudes for their protein-bound and unbound forms (see Section 3.2 for details described there, not for a pixel-wise fit but for a fit of the sum of the decay curves in all pixels). To obtain FLIRR images, the relative amplitudes of bound NADH and FAD were divided for each pixel according to Equation (2).

(2) $$FLIRR=\frac{a_{bound}\left(\mathrm{NADH}\right)}{a_{bound}\left(\mathrm{FAD}\right)}=\frac{a_{2}\left(\mathrm{NADH}\right)}{a_{1}\left(\mathrm{FAD}\right)}$$

**Figure 3:**
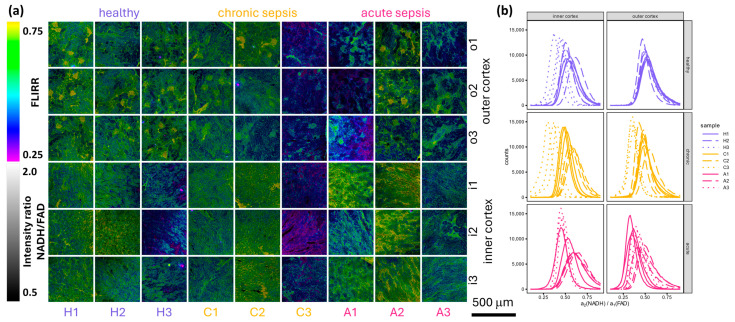
This overview of FLIRR detail images (**a**) and histograms of these images (**b**) shows no considerable differences between samples from healthy mice and those with chronic and acute sepsis. (**a**) The areas of different brightness, which encode the intensity ratio NADH/FAD and show different colors and, therefore, FLIRR are distinguishable. The different areas are those of the proximal and distal tubules. (**b**) The variance between the histograms is higher for the inner cortex positions than for the outer cortex positions, probably due to partial interlacing with renal medulla tissue. Furthermore, the variance between kidneys from different mice is higher for chronic and acute sepsis compared to healthy samples. This may be due to a different immune response between the mice. Sample C3 shows an exceptionally high degree of variation between different positions in the inner cortex, which might be attributed to the high level of damage induced by the comparatively high laser power in these samples, resulting in the formation of new fluorophores. To mitigate these effects in future studies, lower excitation powers and shorter exposure times should be considered. Additionally, alternative autofluorescence correction algorithms may help distinguish genuine metabolic shifts from laser-induced artifacts. This is supported by a considerable increase in the number of photons during the measurement (see Table S3 in the Supplementary Material). A change of FLIRR to lower values in the outer cortex of acutely septic mice can be observed but needs to be quantified.

Figure 3 provides an overview of the combined FLIRR and ratiometric fluorescence intensity maps, accompanied by the corresponding histograms. Figure 3a illustrates the distinction between convoluted proximal and distal tubules in the outer cortex, as identified by their FLIRR and ratiometric NADH/FAD intensity with proximal tubules having a higher FLIRR and being brighter due to their higher NADH content [32,33]. To facilitate comparison, histograms of FLIRR and the relative amplitude $a_{1}$ were generated (see Figure 3b and Figure S1a in the Supplementary Material). Of these values, FLIRR is the most promising for a quick and efficient evaluation because it comprises the relative amplitudes of NADH and FAD in one value. It is already applied for the distinction of cells with differing metabolisms from each other. [22] Therefore, it is used for further analysis. The lifetimes are not expected to change significantly because the system of protein-bound and unbound NADH and FAD stays the same regardless of the disease state, hence their fixation. The ratio of bound and unbound FAD/NADH, reflected by the relative amplitudes, will change with a change in metabolism. The FLIRR value comprises the change of the protein-bound/unbound ratio for both NADH and FAD in one value, facilitating the comparison of different samples. Figure 3b shows that FLIRR changes for some mice, hinting at a change to lower FLIRR in the outer cortex of acute samples compared to healthy and chronic samples.

As shown in Figure 3b, the histograms for healthy outer cortex images show considerable overlap. This is to be expected as the metabolic processes occurring in healthy mice are likely to be comparable. In contrast, the outer cortex images from infected mice show less overlap (see Figure 3b). Except for samples H3 and C3 in the inner cortex, the variation between the three images of a single sample is minimal compared to the variation between samples (see Figure 3b). This observation can be attributed to biological variation in the immune response of the mice. The observed metabolic switch in the outer cortex may reflect its higher vulnerability to early sepsis-induced hypoxia, a hypothesis that aligns with the outer cortex’s proximity to the systemic circulation.

The images of the inner cortex show a greater degree of variation (see Figure 3b), particularly in samples H3 and C3, which can be attributed to medullary entanglement in the cortex. This attribution is reinforced by the overview images in Figure 2, where the squares indicating the position of the detailed images show that, partly, low-fluorescence medulla tissue is included in the measured region. The use of low-resolution preview images with short acquisition times to minimize bleaching did not allow for the identification of a suitable measurement position in the inner cortex for every position of a detailed image, and therefore, not all medullary traces could be excluded. Another reason for the variation for sample C3 in the histogram in Figure 3b is the bright squares of different mean photon arrival times in the overview images of the same sample (see Figure 1). Sample H3, while also showing more variance in the histogram (see Figure 3b), does not exhibit bright tiles in Figure 1 but bright tubular structures.

**Figure 4:**
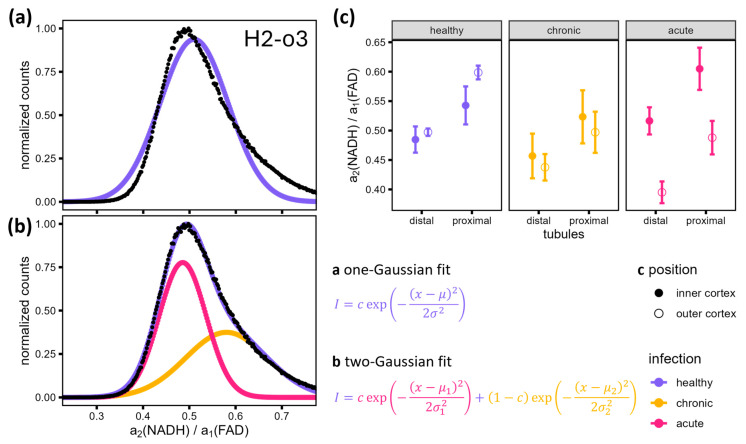
(**a**) The FLIRR histogram of sample H2-o3 (black) with a fit using a single Gaussian curve (violet) shows a deviation from a normal distribution. (**b**) A fit using the sum (violet) of two Gaussian curves (pink, orange) shows that the image comprises two regions, each exhibiting a normal distribution of FLIRR. These two regions can be attributed to the distal ($\mu_{1}$) and proximal tubules ($\mu_{2}$), as visible in Figure 3. (**c**) Comparison of mean fitted FLIRR and its standard error in the distal and proximal tubules of each infection group in the inner and outer cortex shows differences between the inner and outer cortex of kidneys of mice infected with acute sepsis, indicating a metabolism change in this region of the kidneys of these specimens.

For a more detailed comparison of the histograms shown in Figure 3b, it is necessary to examine their key values. If the FLIRR values are normally distributed, a Gaussian fit can be performed. The histogram in Figure 4a shows that this assumption is incorrect. The reason for this discrepancy is that the images in question have two distinct regions, namely the proximal and distal tubules, as previously described and illustrated in Figure 3. A two-Gaussian fit should be able to accommodate this discrepancy and accurately represent the shape of the histogram, as shown in Figure 4b. A full description of the fitting process can be found in Appendix B. In the two regions of the proximal and distal tubules, the fitted amplitudes and FLIRRs are normally distributed.

The mean values of the Gaussian curves $\mu_{1}$ and $\mu_{2}$ can be used to facilitate a comparison of the FLIRR in the distal ($\mu_{1}$) and proximal ($\mu_{2}$) tubules between the different disease states. The plots shown in Figure 4c illustrate the mean of these fitted values for FLIRR with the error bars indicating the standard error of the mean. Scatter plots showing the fitted FLIRR and $a_{1}$ of the individual detail images can be found in Figure S1b (*a*_1_) and S2 in the Supplementary Material. They show that leaving out the outlier samples previously described in this section (H3 and C3; see variation in the histograms in Figure 3b and areas in the overview images in Figure 1), the images of one region (inner/outer cortex) in one infection group (healthy/chronic/acute) show only little variance. This is also reflected in the standard deviation shown in Figure 4c.

The FLIRR of healthy samples has the lowest standard error of the three infection groups and does not differ between the inner and outer cortex for distal tubules (≈0.49). Outer cortex proximal tubules show slightly higher FLIRR (≈0.60) than inner cortex ones (≈0.54). For the chronic infection group, there is also no difference visible between inner and outer cortex distal tubules (FLIRR ≈ 0.45) as well as proximal tubules (FLIRR ≈ 0.51). The error bars show strong overlap for both kinds of tubules. In contrast to these two infection groups, the inner and outer cortex FLIRR vary strongly in the acute infection group with no standard error overlap of the fitted mean values. Here, the FLIRR values are lower in the outer cortex (distal tubule FLIRR ≈ 0.40, proximal tubule FLIRR ≈ 0.49) compared to the inner cortex (distal ≈ 0.52, proximal ≈ 0.60).

The metabolism in both the distal and proximal tubules of the outer cortex in chronic and healthy samples can therefore be assumed to be oxidative phosphorylation, the standard metabolic pathway in healthy tissue [34]. The overall lower values for distal tubules stem from their higher capability to perform glycolysis compared to proximal tubules. In acute samples, the FLIRR of inner cortex tubules stays roughly the same, but a shift in outer cortex tubules FLIRR is visible (see Figure 4c). This is consistent with the lower mean photon arrival times of the outer cortex in Figure 1 and relates to a switch of the metabolism from oxidative phosphorylation to glycolysis [22]. This is in accordance with the metabolic switch observed in tubular cells in the early phase of sepsis-induced AKI [33,35,36]. It is hypothesized that this shift serves to protect the tubules during this acute phase [35].

## 4. Discussion

The results presented in the preceding section show that in the acute stage, at 24 h after sepsis induction, the metabolism is only changed in the outer cortex of the kidney (see Figure 4c). Even in mice showing almost no symptoms (see A2, Table 2), this change in metabolism occurs. The assignment of a specific region where the metabolism is affected was made possible by using FLIM as an imaging technique compared to common biochemical methods, including blood analysis and the analysis of homogenized renal (cortical) tissue, where only cells of a broader region (kidney or renal cortex) can be distinguished by dissection of the tissue prior to homogenization [34,37].

One possible reason for a change of properties closer to the outer perimeter of the kidney, like the mean photon arrival times visible in Figure 1, is freezing. This can be ruled out because it would also have affected the structure of the tissue. Freezing artifacts like cracks and holes can be found throughout all samples or at least in several samples across different infection states (see Figure 1 and detailed images in Figure 3a). While 2P-FLIM provides valuable spatial resolution, the reliance on specific autofluorophores like NADH and FAD limits its applicability to tissues with sufficient endogenous fluorescence. Future studies should explore its integration with complementary techniques for broader applicability. Possible techniques include immunofluorescence microscopy with established biomarkers for sepsis and second harmonic generation to gain more insight into the biochemical constitution of the tissue. Furthermore, the combination with Raman spectroscopy or AI-based image analysis may improve diagnostic sensitivity.

Some overview images show bright squares or bright tubular structures (see Figure 1, samples C3 and H3). The bright squares visible in sample C3 in Figure 1 can be explained by the use of relatively high laser powers for the investigation of this sample. They were required to achieve the desired initial count rate of 250 kcps (see Table S3 in the Supplementary Material) but resulted in photodamage evident by these tiles leading to an elevated fluorescence intensity (see bright tiles in Figure 1) and changes in the relative amplitudes (see Figure S1 in the Supplementary Material). This results in a shift in FLIRR (see Figure 3b). The higher intensity shows that not only (partial) bleaching of the fluorophores occurred, which would also lead to amplitudes but to a reduced fluorescence intensity. A new fluorophore seems to be induced by the high laser power, resulting in a higher fluorescence intensity. The origin of the bright tubular structures in sample H3 (see Figure 1) is unclear but they are most likely the reason for the exceptional behavior of this sample in comparison to the other healthy samples measured with similar excitation laser powers (see FLIRR histograms in Figure 3b and excitation powers in Table S3 in the Supplementary Material). Excluding these two samples, the variance of the amplitudes in the distal and proximal tubules is low (see Figures S1b and S2 in the Supplementary Material). Because of this, the influence of other fluorophores can be ruled out for these samples. The results demonstrate the presence of only NADH and FAD, which is in accordance with the implemented excitation and detection wavelengths as a result of the spectral scan (see Section 3.3 and Figure 2). The reversible metabolic changes observed in the outer cortex suggest that early interventions targeting glycolytic pathways could mitigate sepsis-induced kidney damage, offering new therapeutic avenues. These metabolic shifts are not exclusive to renal tissue. Similar transitions from oxidative phosphorylation to glycolysis have been observed in hepatocytes and immune cells in response to sepsis. Expanding FLIM-based studies to other organ systems could further elucidate the systemic effects of sepsis on cellular metabolism.

In cases of chronic kidney disease, the cells are not able to switch back to oxidative phosphorylation [38]. In the study presented here, chronic refers to the time observation point after induction of sepsis (14 days), regardless of the disease state/score values. These findings based on the FLIRR results visible in Figure 4c show that the switch in metabolism in the outer cortex from oxidative phosphorylation to glycolysis as a reaction to acute sepsis after induction with PCI was reversible. It can be assumed that the mice labeled as chronic here are recovered because their FLIRRs are similar in the inner and outer cortex and close to those of healthy specimen. This fits the lack of symptoms (see Table 2); however, a lack of symptoms alone cannot be used to assign the disease state, as the study of sample A2 showed. The lack of symptoms might imply that the variation of the fluorescence parameters in the outer cortex and therefore the change in metabolism is not induced by the infection but by other influences on the standardized animals. However, the equal treatment of the mice following the infection implies that the observed differences in FLIRR and mean photon arrival times (see Figure 1 and Figure 4c) are a result of the different disease states of the mice. It is also known that infections do not always result in immediate symptoms. Sepsis might be asymptomatic, making it even more important to understand the metabolic pathways to be able to initiate treatment of infected patients as soon as possible [39,40].

## 5. Conclusions

We have demonstrated that 2P-FLIM can effectively differentiate between oxidative phosphorylation and glycolysis in tubular kidney cells from healthy mice and those with acute sepsis. The average photon arrival time overview images revealed distinct differences in the outer and inner cortex of the acute sepsis group, which indicates metabolic changes. Interestingly, not all tubular cells are affected uniformly and at the same time. Initially, 24 h after infection, cells near the outer edge of the kidney exhibit a metabolic shift toward glycolysis, as detected by FLIM and described by FLIRR. However, by 14 days post-infection, this shift reverses, and the metabolism returns to oxidative phosphorylation. This process is visible for the FLIRR of both distal and proximal tubules. The FLIRR histograms were fitted by a two-Gaussian fit and the mean values of the two Gaussians represent the mean FLIRR in the proximal and distal tubules. Another approach to identifying the FLIRR of the two different kinds of tubules would be to use masks to analyze the pixels in the two regions individually. The analysis of the histograms proved to be sufficient but this more complex approach might be of use in the future. To our knowledge, we are the first to report using FLIM during the early stages of sepsis. The metabolism of the outer renal cortex undergoes a reversible transition from oxidative phosphorylation to glycolysis, while the inner cortex remains unchanged. This research establishes a foundation for more detailed FLIM studies on the effects of sepsis and other infections on the metabolism across different regions of various organs. These findings not only enhance our understanding of sepsis pathology but also highlight the potential of FLIM as a diagnostic tool for early therapeutic interventions in septic patients.

## Figures and Tables

**Table 1:** Comparison of different metabolic imaging techniques shows the advantages of 2P-FLIM and its potential for sepsis diagnostics.

Technique	Sensitivity to Metabolism	Label-Free?	Resolution	Clinical Applicability
2P-FLIM	High (FLIRR enables quantitative analysis)	Yes	High (subcellular)	Potential for sepsis diagnostics
Raman spectroscopy	Medium	Yes	Medium	Tissue characterization
Immuno- fluorescence	High	No	High	Marker-specific diagnostics

**Table 2:** Behavioral scores attributed to the mice at different timepoints after the infection. In the following timepoints, all scores were zero (96+).

Infection	Sample	Average Score	Time After Infection/h													
			6	12	18	24	30	36	42	48	54	60	66	72	84	96+
Healthy *	H1	0												0		0
	H2	0												0		0
	H3	0												0		0
Chronic Sepsis	C1	3.55	4	7	7	7	5	3	3	2	0	0	1	0	0	0
	C2	4.36	5	6	7	7	6	4	5	4	1	1	2	0	0	0
	C3	4.44	4	6	7	7	6	3	3	3	1	0	0	0	0	0
Acute Sepsis	A1	7.50	5	8	8	9										
	A2	1.00	1	1	0	0										
	A3	7.75	6	8	8	9										

* Healthy mice scored every three days for comparison.

**Table 3:** Results of the spectral FLIM scan of a healthy sample (mouse H2): selected excitation ($\lambda_{ex}$) and detection ($\lambda_{det}$) wavelengths, as well as approximate lifetimes and relative amplitudes for NADH and FAD. The lifetimes $\tau_{1}$ and $\tau_{2}$ were fixed at these values for the pixel-wise fits of the detailed images presented in Section 3.4.

Fluorophore	*λ_ex_*/nm	*λ_det_*/nm	*a*_1_/%	*τ*_1_/ps	*τ*_2_/ps
FAD	876	550–650	66.1	400	2650
NADH	711	380–450	63.8	420	2170

## Data Availability

Dataset available on request from the authors.

## Supplementary Materials

The following supporting information can be downloaded at: www.mdpi.com/article/10.3390/bioengineering12020170/s1, Table S1: Behavioral Score Calculation Parameters; Table S2: Overview Images Parameters including the size of the overview image in tiles, the laser pulse width Δ*λ* and laser power at the sample *P_s_*; Table S3: Detailed image parameters of healthy, chronic and acute samples with the laser pulse width Δ*λ*, final count rate after the measurement *R_f_* and laser power at the sample *P_s_*. The positions o1, o2, o3 are located in the outer cortex and i1, i2 and i3 in the inner cortex close to the medulla; Figure S1: Histograms of $a_{1}$ parameter maps (a) and means of the two fitted Gaussian curves (b) showing differences between the relative amplitudes $a_{1}$ in the inner and outer cortex, which are leading to differences in FLIRR values; Figure S2: Means of the two fitted Gaussian curves for FLIRR parameter maps show that in both proximal and distal tubules that FLIRR varies between inner and outer cortex acutely septic samples.

## Appendix A

Unless otherwise stated in this section, the following common image parameters were used for both overview and detail images. Images of 1024 × 1024 px with 1× zoom resulting in a field of view of (581.25 μm)^2^ were obtained with a scan rate of 600 Hz and a fully open pinhole (10.60 AU (580 nm)).

The varying parameters for the detail images are shown in Table S3 in the Supplementary Material. For the overview images, the tuneable (HyD-SMD 1, 2P-FLIM) detector was set to 455–510 nm. Images were acquired using a combined excitation with the pump laser at 798 nm and a second laser at 1032.6 nm. The laser powers and dimensions of the overview images are given in Table S2 in the Supplementary Material. The focus was adjusted at a minimum of five points to obtain a focus map. Twelve frame accumulations were performed. An exception to the common parameters mentioned above was sample C1, where a resolution of 512 × 512 px was used. Overview images were merged using the ImageJ (version 2.14.0) plugin Grid/collection Stitching [41].

The use of a measured instrument response function (IRF) is preferable to that of a fitted IRF [42]. The IRF was measured using crushed sugar in a glass-bottom dish, which was covered with a piece of black cardboard in order to limit reflexes. Due to the time in between measurements, different IRFs were utilized for each measurement. For all three IRFs, an excitation wavelength of 876 nm and a detection window between 428 and 448 nm were employed. The specific measurement parameters are outlined in Table A1. All other parameters remained consistent with those utilized for the detailed FLIM images, except for the 48x zoom and a resolution of 256 px × 256 px.

**Table A1:** Measurement parameters for IRF measurements with laser pulse width Δλ, laser power at the sample $P_{s}$, and frame rotation θ.

Samples	*Δλ*/nm	*P_s_*/mW	*θ*/°	Accumulations
H1. H2, H3, C1, C2, C3	0.7	27	100	1000
spectral scan	0.9	27	100	2000
A1, A2, A3	0.7	19	3	1000

All laser powers mentioned were calculated using a linear conversion of the measured laser power at the laser output using an optical power meter on the objective (PM400USB, ThorLabs, Newton, NJ, USA). The parameters used were consistent with those utilized for the detailed FLIM measurements, except for a resolution of 512 × 512 px and 48× zoom.

## Appendix B

It is assumed that the fluorescence parameters $a_{1}$ and FLIRR, resulting from the fitting of the decay curve, are normally distributed in each of the two regions visible in the detail images, namely convoluted proximal and distal tubules. Consequently, a fit using the sum of two Gaussian curves can be employed to describe the histograms. To obtain a more robust fit using two Gaussian curves, an initial fit using one Gaussian curve was employed using Equation (A1) and the starting parameters shown in Table A2. These results were then used as the starting parameters for a fit using two Gaussian curves according to Equation (A2) and the parameters shown in Table A3. Both fits were performed using a Levenberg–Marquardt algorithm implemented in R using the minipack.lm package [43]. If $\mu_{2}$ was smaller than $\mu_{1}$, the respective values were swapped and c was set to 1−c.

(A1) $$N=a\;e^{-\frac{{\left(x-\mu \right)}^{2}}{2\sigma^{2}}}$$

(A2) $$N=a\;\left(c\;e^{-\frac{{\left(x-\mu_{1}\right)}^{2}}{2\sigma_{1}^{2}}}+\left(1-c\right)\;e^{-\frac{{\left(x-\mu_{2}\right)}^{2}}{2\sigma_{2}^{2}}}\right)$$

**Table A2:** Starting parameters of one Gaussian fit.

	a			μ			σ		
Parameter	Min	Max	Start	Min	Max	Start	Min	Max	Start
$a_{1}$	0.5	1	0.95	60	90	65	1	20	5
FLIRR	0.5	1	0.95	0.25	0.75	0.5	0.01	0.4	0.08

**Table A3:** Starting parameters for the fit using two gaussian curves a, c, $\mu_{1}$, $\mu_{2}$, $\sigma_{1}$, $\sigma_{2}$, depending on the results of the fit using one gaussian curve (*μ*, *σ*).

	a	c	$\mu_{1}$ $,\;\mu_{2}$	$\sigma_{1}$ $,\;\sigma_{2}$
min	0.8	0.0	*μ*-2*σ*	0.3 *σ*
max	1.3	1.0	*μ*+2*σ*	3.0 *σ*
start	1.0	0.5	*μ* (FLIRR) 0.95*μ*, 1.05*μ* ($a_{1}$)	*σ*
