# Supplementary material for: Characterizing Metabolic Shifts in Septic Murine Kidney Tissue Using 2P-FLIM for Early Sepsis Detection
Source: Bioengineering (Basel). 2025 Feb 10;12(2):170. doi: 10.3390/bioengineering12020170 (PMC11851710; doi:10.3390/bioengineering12020170)
Supplement: Supplementary file 1 [file bioengineering-12-00170-s001.zip › bioengineering-3396460-supplementary.pdf]

Table S1. Behavioural Score Calculation Parameters.

Score Item	Score	Phenotype
Body Weight	0	unchanged/similar weight
	2	reduction of weight between 11-20%
	3	reduction of weight above 20%
General condition	0	Smooth coat, body opening, clear eyes
	1	decreased maintaining hygiene, temporary eye/nasal discharge, hair loss, diarrhoea, slightly curved
	2	coat dull, eye cloudy or glued, body closed, curved
	3	unkempt, shaggy, or dirty fur, loss of grip strength, tremor, convulsions, severely hunched over
activity	0	locomotion, curiosity, social
	1	low activity, frequent sleep, occasional motion disruption
	2	slow movement, drowsy
	3	lethargy, no movement
reaction to external stimuli	0	curious, fast movement
	1	reduced attention yet timely adequate response
	2	reduced and delayed response
	3	no response

Table S2. Overview Images Parameters including the size of the overview image in tiles, the laser pulse width $\Delta\lambda$, and laser power at the sample P_s.

			876 nm		1032 nm
**Sample**	**Columns**	**Rows**	P_s **/ mW**	$\Delta\lambda$ **/ nm**	P_s **/ mW**
H1	15	13	52	0.6	29
H2	15	17	52	0.7	30
H3	15	13	52	0.6	29
C1	13	15	52	0.7	29
C2	10	12	53	0.7	29
C3	14	13	51	0.8	29
A1	13	15	37	0.8	26
A2	15	11	36	0.8	24
A3	18	15	34	0.7	22

Table S3. Detail image parameters of healthy, chronic and acute samples with the laser pulse width $\Delta\lambda$, final count rate after the measurement R_f and laser power at the sample P_s. The positions o1, o2, o3 are located in the outer cortex and i1, i2 and i3 in the inner cortex close to the medulla.

			λ_{ex}= 876 nm			λ_{ex}= 711 nm	
**Sample**	**Position**	$\Delta\lambda$ **/ nm**	R_f **/ kcps**	P_s **/ mW**	$\Delta\lambda$ **/ nm**	R_f **/ kcps**	P_s **/ mW**
H1	o1	0.6	190	42	0.9	235	54
	o2	0.5	220	45	0.8	230	55
	o3	0.6	235	36	0.9	235	50
	i1	0.6	225	33	0.8	230	43
	i2	0.7	230	33	0.8	230	44
	i3	0.7	220	33	0.9	230	43
H2	o1	0.6	230	40	0.8	220	55
	o2	0.7	225	37	0.8	220	52
	o3	0.6	215	41	0.8	225	54
	i1	0.7	210	46	0.8	220	51
	i2	0.8	225	49	0.8	220	52

	i3	0.7	220	45	0.8	230	55
H3	o1	0.6	235	41	0.8	220	52
	o2	0.7	225	34	0.8	215	51
	o3	0.7	220	40	0.7	220	54
	i1	0.8	220	48	0.8	220	51
	i2	0.6	220	43	0.7	220	51
	i3	0.8	230	46	0.8	230	51
C1	o1	0.6	235	37	0.8	235	58
	o2	0.6	240	38	0.8	230	55
	o3	0.5	240	36	0.8	245	50
	i1	0.7	230	35	0.8	230	47
	i2	0.6	225	38	0.8	230	50
	i3	0.6	220	37	0.8	220	47
C2	o1	0.6	240	32	0.8	230	53
	o2	0.6	235	28	0.8	235	43
	o3	0.6	230	32	0.8	225	52
	i1	0.6	230	39	0.8	230	48
	i2	0.6	230	41	0.9	230	52
	i3	0.6	220	41	0.8	220	50
C3	o1	0.7	205	48	0.8	230	83
	o2	0.8	225	40	0.7	220	77
	o3	0.8	220	39	0.8	210	73
	i1	0.8	220	47	0.7	220	75
	i2	0.6	200	56	0.7	225	83
	i3	0.7	225	49	0.7	225	75
A1	o1	0.6	240	19	0.9	230	30
	o2	0.8	230	18	0.8	240	33
	o3	0.8	230	19	0.8	270	33
	i1	0.8	230	19	0.9	225	23
	i2	0.7	225	20	0.9	220	25
	i3	0.7	220	19	0.9	220	24
A2	o1	0.6	215	23	0.9	230	34
	o2	0.7	215	19	0.8	205	32
	o3	0.7	230	21	0.8	230	34
	i1	0.6	225	24	0.8	200	26
	i2	0.8	215	19	0.8	220	24
	i3	0.8	225	20	0.8	215	25
A3	o1	0.7	235	18	0.9	220	36
	o2	0.8	230	15	0.9	215	31
	o3	0.8	235	15	0.9	230	29
	i1	0.7	220	22	0.9	220	28
	i2	0.8	230	18	0.9	210	24
	i3	0.8	230	15	0.9	220	26

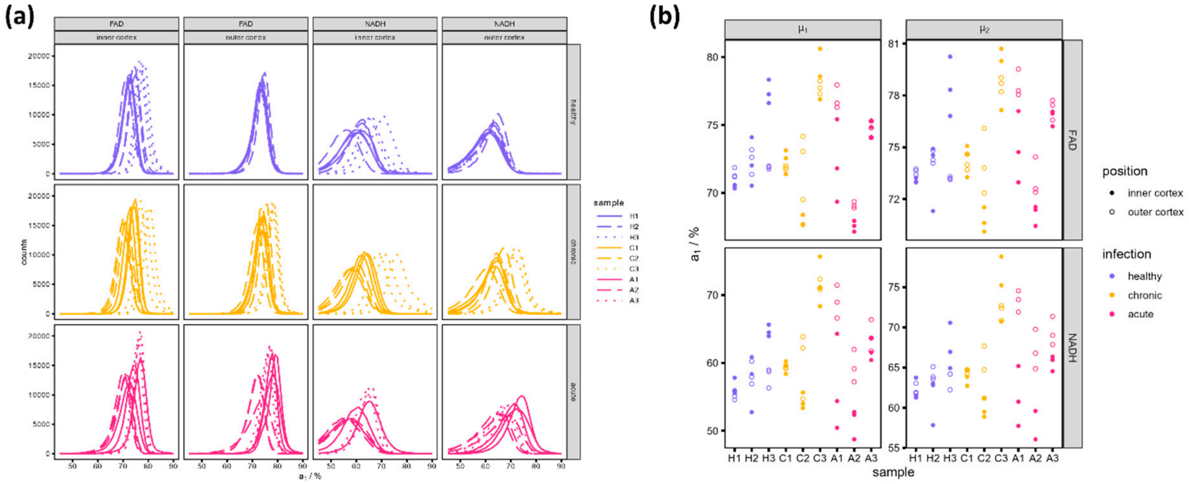

Figure S1. Histograms of a_1 parameter maps (a) and means of the two fitted Gaussian curves (b) showing differences between the relative amplitudes a_1 in the inner and outer cortex, which are leading to differences in FLIRR values.

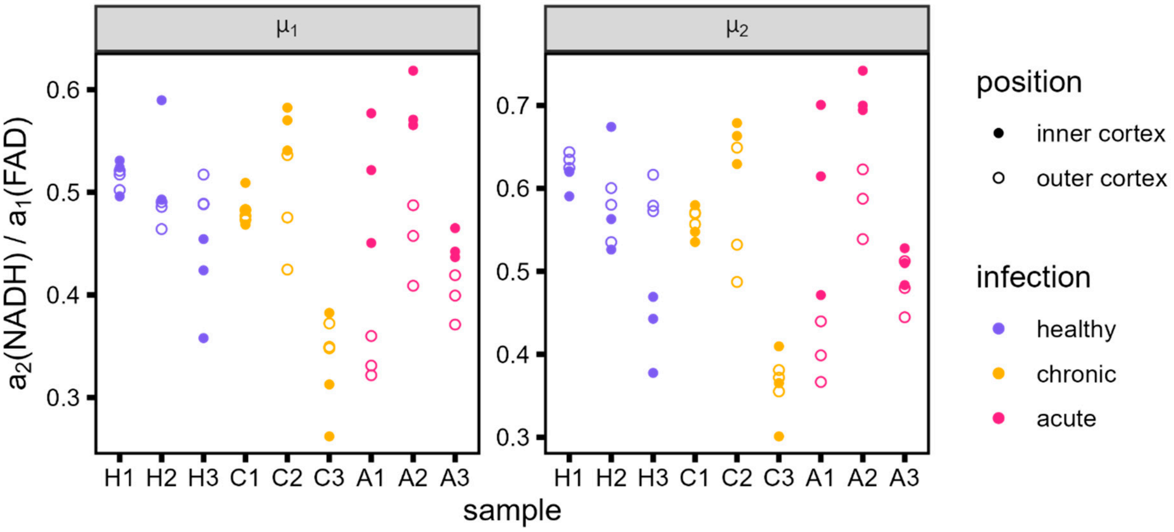

Figure S2. Mean values of the two fitted Gaussian curves for FLIRR parameter maps show that in both proximal and distal tubules that FLIRR varies between inner and outer cortex acutely septic samples.
